# Vaccination With Sporozoites: Models and Correlates of Protection

**DOI:** 10.3389/fimmu.2019.01227

**Published:** 2019-06-05

**Authors:** Yun Shan Goh, Daniel McGuire, Laurent Rénia

**Affiliations:** ^1^Singapore Immunology Network (SIgN), Agency for Science, Technology and Research (A^*^STAR), Biopolis, Singapore, Singapore; ^2^School of Biological Sciences, Nanyang Technological University, Singapore, Singapore; ^3^Department of Microbiology and Immunology, Yong Loo Lin School of Medicine, National University of Singapore, Singapore, Singapore

**Keywords:** *Plasmodium falciparum*, malaria, sporozoites, protection, vaccine, models, correlates

## Abstract

Despite continuous efforts, the century-old goal of eradicating malaria still remains. Multiple control interventions need to be in place simultaneously to achieve this goal. In addition to effective control measures, drug therapies and insecticides, vaccines are critical to reduce mortality and morbidity. Hence, there are numerous studies investigating various malaria vaccine candidates. Most of the malaria vaccine candidates are subunit vaccines. However, they have shown limited efficacy in Phase II and III studies. To date, only whole parasite formulations have been shown to induce sterile immunity in human. In this article, we review and discuss the recent developments in vaccination with sporozoites and the mechanisms of protection involved.

## Introduction

Malaria is one of the deadliest diseases, causing a major public health problem with high mortality and morbidity. In 2017, the World Health Organization reported 219 million clinical cases and 435,000 deaths (1). The use of different control interventions such as insecticide-treated bed nets, combination drug therapies and early diagnostics has greatly reduced malaria mortality worldwide (2). However, with increasing drug resistance and insecticide resistance, these efforts are insufficient to eradicate malaria globally (3, 4). It has become increasingly clear that there is no control intervention that can singly eradicate malaria. Multiple control interventions need to be in place simultaneously and a malaria vaccine is integral to global malaria eradication (5).

## Parasite Life Cycle

*Plasmodium* parasites have a complex life cycle, infecting two hosts, the human and the mosquito. In the human host, the *Plasmodium* life cycle consists of two stages, the liver stage and the blood stage. Infected female *Anopheles* mosquitoes inject sporozoites into the dermis of their mammalian host upon feeding. Sporozoites then enter the bloodstream and migrate to the liver, where the liver stage begins. The sporozoites multiply in hepatocytes, eventually forming merozoites in vesicles called merosomes. These vesicles rupture and release the merozoites into the bloodstream, commencing the blood stage by infecting red blood cells (RBCs). It is the continual cycling of malaria parasites within the RBCs, and the immune responses directed against this stage of the parasite, that causes most of the pathologies observed in malaria infections. The malaria parasites are then transmitted back to the mosquito following blood feeding by a female mosquito. The sexual forms of the blood stage parasites, gametocytes, develop into male and female gametes which fertilize each other, eventually forming oocysts in the mosquito's midgut wall. The oocysts then lyse to release sporozoites, which migrate to the mosquito's salivary glands. When the *Anopheles* mosquito takes a blood meal on another human, the injected sporozoites migrate from the dermis to the liver, thereby beginning a new cycle of infection.

## Vaccines Against Malaria

The development of vaccines for malaria has been met with many difficulties. Despite decades of research efforts, there is still no available vaccine for human use. This has led to the development of a wide range of approaches, in the search for an efficacious malaria vaccine. These approaches can be broadly divided into three main categories: (1) whole parasite-based vaccines, (2) subunit vaccines, and (3) viral, bacterial and parasite vectors as delivery vectors.

### Whole Parasite-Based Vaccines

Whole parasite-based vaccines have had considerably more success than other vaccines. Whole parasite-based vaccines contain all parasitic antigens. This approach allows the development of different types of immune responses. Whole parasites used for the vaccines are obtained by dissecting sporozoites from mosquitoes or harvesting asexual blood stages from culture. There are many technical, logistical, and regulatory hurdles associated with large scale production and delivery of whole parasite vaccines in the field. However, recent sporozoite vaccine trials have shown considerable progress in overcoming these hurdles (6, 7).

#### History

The development of malaria vaccines began with whole parasite-based vaccines more than a 100 years ago when the Sergent brothers used heat-inactivated *P. relictum* sporozoites to immunize canaries and obtained partial protection (8). This was followed by the work of Russell and Mohan where both cellular and humoral responses against malaria were induced in immunized domestic fowls (9). In 1946, Jules Freund invented the Freund adjuvant and formulated the vaccine by combining the adjuvant with formalin-inactivated-blood infected with *P. lophurae*, an avian malaria parasite, or *P. knowlesi*, a monkey malaria parasite (10, 11). The formulations showed promising efficacy. However, the toxic side effects of the Freund adjuvant have prevented its use in humans. The first attempt in humans was done by Heidelberger et al., using formalin-inactivated *P. vivax*-infected blood to immunize volunteers, however no protection was induced (12). These initial studies, though suboptimal, have paved the way for future whole parasite-based vaccine development.

#### Vaccination With Sporozoites

Among the whole parasite-based vaccine candidates, there is considerable research on the pre-erythrocytic parasites. The pre-erythrocytic stage is an asymptomatic phase. Very few sporozoites are injected and subsequently developed in the hepatocytes during natural infection in human volunteers (13). The idea of inducing an immune response that can neutralize sporozoites in the skin and circulation and prevent the penetration of a low number of sporozoites into hepatocytes or destroy a low number of infected hepatocytes during the asymptomatic phase make pre-erythrocytic stage vaccines attractive. By preventing the pre-erythrocytic stage development, the vaccines would prevent blood stage infection, hence preventing pathology. In addition, the pre-erythrocytic stage vaccines have had more success than the other stages, which provides more support for their development.

Whole sporozoite-based vaccines developed thus far in human include (1) irradiated parasites (6, 14), (2) genetically-attenuated parasites (15, 16), and (3) drug-infection-treatment vaccination (17, 18).

##### Vaccination with irradiated sporozoites

Irradiated sporozoite vaccine is the most clinically developed whole parasite-based vaccine, and also the most clinically developed pre-erythrocytic vaccine (17). The first few studies that showed definitive protective immunity with irradiated sporozoites were done in chicks and subsequently in mice (18, 19). This was later demonstrated in humans, where vaccination with irradiated sporozoites, *via* infective mosquito bites, protected 92% of the volunteers from infection (20–22). However, >1,000 mosquito bites are required to introduce sufficient irradiated sporozoites to induce the high level of efficacy. This prevented the development of this approach for mass vaccination.

More recently, delivery of cyropreserved irradiated sporozoites into the host by direct venous inoculation *via* needle and syringe, has been tested in humans and showed promising efficacy data (6, 17, 23). While four doses only protected 33% of the individuals (6), five doses protected 100% of the individuals (6). More studies to perfect the vaccination regimes would allow direct venous inoculation *via* needle and syringe to replace mosquito bites as a delivery system. Another hurdle with irradiated sporozoite vaccines is the need for a high dose of irradiated parasites. Vaccine dosage, vaccination regimen, and route of administration have been investigated in malaria-naive adults (24). In the study, administration of higher doses may further enhance protection— four intravenous immunizations with a higher dose of 2.7 × 10^5^ irradiated sporozoites was found to be the most optimal, where 55% of vaccinated subjects remained uninfected following controlled human malaria infections (CHMI) 21 weeks after immunization. The timing of the CHMI following vaccination has also been found to be important, with vaccine efficacy being higher when CHMI was performed 3 weeks after immunization, instead of 21 weeks. While vaccination with irradiated sporozoites led to sterile protection in 100% (6/6) of vaccinated malaria naïve volunteers (6), irradiated sporozoites vaccination in malaria-endemic Mali yielded a lower protection (14). There are fundamental differences between the two studies, such as the first study examines protection against homologous challenge and the latter study examines protection against heterologous challenge. Naturally transmitted parasites are often different from the vaccine strain. Twenty-four weeks after final immunization regimen, five doses of 2.7 × 10^5^ irradiated sporozoites protected 7 of 10 against homologous CHMI, but only 1 of 10 against heterologous CHMI (25), showing that the vaccine efficacy against heterologous infection is markedly reduced. In addition, the findings also suggest that pre-exposure to the malaria parasites may prevent the establishment of a protective immune response since blood stage infection is able to induce immune suppression (26). This also further highlights the need for optimization of the required dose and regime in the target population to achieve robust and sterile protection.

The irradiation of parasites is a delicate process that requires the sporozoites to retain a certain degree of viability. Similar to heat-inactivated and frozen-thawed sporozoites, over-irradiated sporozoites do not induce protection (27, 28). Irradiating the sporozoites leads to DNA damage in the sporozoites with no or limited reduction on hepatocyte infectivity (29, 30). Although irradiation results in an inhibition of parasite DNA replication, ultrastructure modification and alteration in gene expression (31, 32), eventually leading to developmental arrest of the liver stage within hepatocytes (33, 34), it still allows for parasite antigen presentation and priming of immune responses.

##### Vaccination with genetically-attenuated sporozoites

Non-irradiated sporozoites have been postulated to be more efficacious as whole parasite-based vaccines because, as compared to irradiated sporozoites, they are able to progress to a later stage of pre-erythrocytic development, (35). By doing so, the host immune system is exposed to a wide repertoire of malarial antigens and thus able to target more of the pre-erythrocytic stage. To this end, research efforts have focused on developing ways, other than irradiation, to attenuate the growth of the parasite. Recent advances in *Plasmodium* research such as genetic manipulation have brought forward a new approach to attenuate parasites. Genetically-attenuated parasites are modified by deleting key essential genes that result in developmental arrest of the liver stage after hepatocyte infection, but do not affect parasite viability, mosquito infectivity, and sporozoite production in animal models (36). Inactivation of UIS3, UIS4, or P36p prevented the attenuated parasites from developing beyond the early pre-erythrocytic stage in mice (37–39). Vaccination of these live genetically-attenuated parasites offered sterile protection against a challenge with a wild type isolate. While promising, one of the major concerns for the development of genetically-attenuated parasites as vaccines is the possible occurrence of breakthrough infections. The first clinical trial using live genetically-attenuated parasites that lack the two genes, p52 and p36, led to breakthrough infections (15). Breakthrough infections in mice have also been observed for UIS4- and P36p-deficient parasites (37, 39). However, great progress has been made recently. Live genetically-attenuated parasites lacking three genes (p52–/p36–/sap1–; “PfGAP3KO”) arrest early in liver-stage development, and were safe with no observed breakthrough infection following administration into human subjects by infective mosquito bites (16). Compared to irradiated sporozoite vaccine and early liver stage-arresting genetically-attenuated parasites, second generation genetically-attenuated parasites that arrest late liver stages have shown to demonstrate superior anti-malarial immunity following vaccination in mice by having a greater antigen repertoire (40, 41). These candidate vaccines could also have greater efficacy in humans, but this remains to be demonstrated.

##### Drug-infection-treatment vaccination

Drug-infection-treatment vaccination is the last approach. It involves vaccination with live wild-type parasites under drug prophylaxis, where the drug targets and eliminates the blood stage parasites. This approach allows full liver development and a limited initial blood stage development, thereby focusing immunity toward the liver stages. Pioneer mouse studies have, indeed, shown greater efficacy when the mice were vaccinated with live *P. berghei* or *P. yoelii* sporozoites under drug prophylaxis than when the mice were vaccinated with irradiated sporozoites—fewer inoculations and less sporozoites were required to induce sterile protection (42–44). Vaccination with live sporozoites under chloroquine prophylaxis is the most investigated vaccine formulation under this approach, and it has shown very promising efficacy data. The first study in humans examined the efficacy of the vaccine where the live sporozoites were introduced into the volunteers *via* infective mosquito bites (45). The study utilized CHMI and demonstrated sterile protection, where 100% of the volunteers were protected from infection following a wild-type sporozoite challenge. A subsequent CHMI study investigated the efficacy of the vaccine where the live sporozoites were intravenously inoculated *via* a needle and syringe. In this study, a dose-dependent protection was observed, where only three doses of 5.12 × 10^4^ sporozoites were sufficient to protect all volunteers from the challenge (46). In addition to chloroquine, vaccination with live wild-type parasites under prophylaxis of other antimalarials has also been investigated. Primaquine (47), mefloquine (48) and artemisinin derivatives such as artesunate (49) have been used in place of chloroquine and vaccination with live wild-type parasites under prophylaxis of these antimalarials has demonstrated protective immunity against a homologous sporozoite challenge in mice and in humans.

However, there are various challenges with this approach such as the technical and logistical issues associated with generating sporozoites at large scale and field deployment. There are also concerns that the sporozoite injections might not be properly followed with antimalarial treatment, which could lead to sickness. In addition, while sterile immunity can be achieved against a homologous sporozoite challenge, protection was suboptimal when immunized volunteers were challenged with a heterologous strain (50, 51), suggesting that the protective immune mechanisms target polymorphic antigens. Nevertheless, the current published findings have demonstrated very encouraging efficacy data and suggest that vaccination with live sporozoites under chloroquine prophylaxis, following vaccination regime optimization, could potentially be the most efficacious sporozoite vaccine until date.

### Subunit Vaccines

Subunit vaccines allow rational design of the vaccine to induce the desired immune effectors against the parasite. In addition, subunit vaccines are safe and generally easy to produce at large scale and to administer in the field. Hence, efficacious subunit vaccines that offer long term protection are the preferred vaccines of choice.

#### Peptides and Recombinant Proteins

Subunit vaccines have been developed either as peptides, multi-peptide constructs or recombinant proteins. They can be based on a single parasite antigen or a combination of multiple parasite antigens, and often in a formulation that includes adjuvants. Most constructs have been designed for the circumsporozoite protein (CSP), a major sporozoite surface protein (52), as it is the first cloned malaria antigen (53, 54). Both peptides and multi-peptide constructs containing either the B epitope alone or both B and T epitopes induced protection in mouse models (55–58). However, when tested in humans, the peptide constructs did not induce significant protection (59). The main reason for the failure in humans is that the immune response to these constructs was genetically restricted by major histocompatibility gene (60) and thus could not induce an efficient immune response in most volunteers.

To ameliorate immunogenicity and protective efficacy, peptides and proteins have been designed to contain T cell epitopes from the parasites or an unrelated proteins recognized by many MHC genes (61), coupled to diverse chemical backbones, or fused with other proteins to create particle vaccines to be used with or without various adjuvants (62–67). All these new constructs demonstrated a high efficacy in mouse models but have yet to be validated in humans.

Of all the subunit vaccines, RTS,S, a CSP-based subunit vaccine, is the current most clinically-advanced malaria vaccine, being the only malaria vaccine to have progressed to the pivotal Phase III clinical trials (68). Enormous resources have been spent on rationally improving RTS,S efficacy, which include developing novel adjuvant systems. Multiple studies have demonstrated a need for RTS,S be formulated with adjuvants such as monophosphoryl lipid A and QS21, to achieve immunogenicity (69–71). This was followed up by a series of clinical trials, where significant progress has been made to improve immunogenicity and efficacy (72, 73). The RTS,S vaccine has been designed to inhibit the liver stage and prevent blood stage infection. RTS,S is, ultimately, formulated with a chimeric molecule based on CSP, fused to the S antigen of the hepatitis B virus, together with a potent adjuvant, AS01. The first Phase IIb trials performed in adult volunteers in the USA showed ~50% protection against clinical malaria (69, 70, 74).

When tested in the endemic regions, RTS,S's efficacy against infection was less impressive and was of short-duration (<3 months) (75, 76). What is encouraging is that RTS,S/AS01 vaccination has been found to induce a significant reduction (~60%) in the incidence of clinical infections in children in the same study (76). This finding encouraged GlaxoSmithKline and the Malaria Vaccine initiative, with financial support from the Bill and Melinda Gates Foundation, to further develop this vaccine for infants and young children in Africa. However, RTS,S efficacy against clinical malaria was later found to be suboptimal in malaria-endemic populations, with a vaccine efficacy against clinical infection of 36.3% in young children and 25.9% in infants (77–81). One possible explanation is that the CSP used in the vaccine contains several T cell epitopes, which are all highly polymorphic in parasite population in the field. Neafsey et al. elegantly demonstrated that the overall vaccine efficacy was very low in field settings with minimal matching of the CSP alleles in the field with the CSP allele in RTS,S (80).

Another explanation for the limited efficacy of the CSP-based vaccines is that it may not be the best antigen to induce protection. It is likely that other antigens may be better vaccine candidates, alone or in combination with the CSP. This hypothesis was supported by various studies, which showed that the sterile protection against a sporozoite challenge obtained in mice immunized with irradiated sporozoites was independent of the immune response against the CSP (82–85).

With the limited success of subunit vaccines developed thus far, there have been many efforts to identify new pre-erythrocytic (liver) targets for vaccine development using various approaches (86–90). These new antigens have shown encouraging efficacy data in animal models either alone (91, 92) or in combinations (93, 94), however efficacy in humans has yet to be demonstrated (95).

#### DNA Vaccines

DNA has been identified as a vaccine delivery system in the 1990s (96). This approach was quickly taken up, and DNA vaccines against CSP were developed and tested in human (97–99). Although the CSP-based constructs induced high levels of protection in mice (100), they had poor immunogenicity in humans (101). DNA constructs encoding multiple genes (102, 103) or epitopes were also developed (104). However, none of these constructs induced high level of protection against a sporozoite challenge (104, 105). To enhance the immune responses, malaria DNA vaccines were developed in combination with DNA constructs encoding for cytokines such as GM-CSF. Although some of these constructs had increased immunogenicity and efficacy in murine models (106), they did not induce protection against sporozoite challenge in human (105).

### Viral, Bacterial, and Parasite Vectors as Delivery Vectors

Viral, bacterial and parasite vectors have been developed as delivery vectors for malaria vaccines. As these vectors are based on whole organisms, they usually do not need to be adjuvanted to stimulate the innate immune system which is necessary for the development of an optimal adaptive immune response (107). However, in some studies, various adjuvants have also been used to increase vector constructs immunogenicity (108).

The use of viral vectors as delivery vectors for malaria vaccines is the most common (109). Recombinant influenza viruses (110), pox viruses such as vaccinia virus (110, 111), Sindbis virus (112), yellow fever virus (113, 114), adenovirus (115), human cytomegalovirus (116) as delivery vectors have shown promising efficacy in animal models. Bacterial vectors such as *Salmonella* (117, 118), Bacille-Calmette Guerin (BCG) (119), *Shigella flexneri* 2A strain (120) as delivery vectors have also shown good immunogenicity and efficacy against sporozoite challenge in mice. Currently, only *Salmonella* vectors as delivery vectors have been examined in humans, showing good safety profile and immunogenicity (121). Parasites such as *Leishmania* (122) and *Toxoplasma* (123, 124) as delivery vectors were also able to induce partial protection in mice. However, it remains to be seen if these vectors can induce protection in human.

### Prime-Boost Combinations

To enhance humoral and T cell responses, various prime-boost strategies have been developed using combinations of different vaccine approaches. Vaccination with subunits or DNA constructs, followed by viral vectors or combination of viral vectors encoding one or multiple malaria antigens has been examined and efficacy has been demonstrated in mouse models (125–129). However, of all these combinations, only a few have shown significant efficacy in humans (104, 109, 130–133). Recent strategies, prime-and-target (134) and prime-and-trap (135), have shown that the best combination, that can induce high level of protection in mice, depends on the capacity to induce and maintain tissue-resident memory cells in the liver.

## Model Systems

The use of experimental models has an important role in the development of vaccines. It is essential for the first assessments of safety, immunogenicity and potential protective efficacy of vaccine candidates. The early studies of malaria candidate vaccines utilized avian models, despite being a poor alternative to study pathogens with mammalian hosts. The eventual establishment of other malaria models has brought new insights and greatly facilitated malaria vaccine research. Current models used include: (1) mouse, (2) non-human primates (NHP), (3) humanized mice, and (4) human volunteers (CHMI).

### Mouse Models

Until date, the traditional mouse model still remains the most commonly used model as it is less costly and more easily available. *P. berghei* and *P. yoelii* are two of the more commonly used rodent malaria species for *in vivo* and *in vitro* studies. It is often the starting ground for *in vivo* studies examining the development of pre-erythrocytic stage and whole sporozoite vaccine candidates. Human parasites such as *P. falciparum* and *P. vivax*, which contribute to the majority of the malaria global disease burden, display highly restricted host-cell tropism—they cannot establish an infection and develop the pre-erythrocytic stages effectively *in vitro* easily. A reproducible model of full development of the pre-erythrocytic stage has only been described in primary human hepatocytes (136). The mouse model allows the examination of the parasite infection in the liver *in vivo*, and the effectiveness of the whole sporozoite vaccine candidates to protect the host from infection. While vaccine efficacy in the mice does not necessarily predict vaccine efficacy in humans, there have not been any examples where an absence of vaccine efficacy in mice was contradicted by vaccine efficacy in humans. Quite a number of whole sporozoite vaccine candidates that are first identified to be protective in mouse models (19, 42, 44) have went on to be validated in humans (22, 45). The first study demonstrating that vaccination with a whole sporozoite vaccine candidate, irradiated sporozoites, can induce sterile protection from infective sporozoite challenge was in a *P. berghei* mouse model (19). This has been established as the gold standard as human volunteers vaccinated with irradiated *P. falciparum* sporozoites were found to develop protective immunity (22). Similarly, vaccination with live sporozoites under drug prophylaxis was also first identified to have promising efficacy in the *P. berghei* and *P. yoelii* mouse models (42, 44), before demonstrating sterile protection in all vaccinated human volunteers (45). A new approach to chemically attenuate sporozoites has been identified using the *P. berghei* model. This was performed by treating sporozoites with centanamycin, a DNA alkylating agent (137–139). This may also offer protection in humans, however further studies to validate its efficacy are still pending.

With the development of transgenic rodent malaria parasites, knock-in (KI) parasites expressing *P. falciparum* (140) or *P. vivax* (141) genes have been generated. These KI parasites allow the examination of the efficacy of immunogens (142–144) or antibodies against human malaria pre-erythrocytic antigens *in vivo* (145, 146).

While the mouse models have greatly contributed to the development of malaria vaccines, there are limitations. It is still largely unknown how relevant the mouse models are for the human parasite. The ability to interpret and draw conclusion from the mouse and translate it to the human remains unclear. There are major fundamental differences, both at genetic and proteomic levels, between the rodent and human malaria species, with the rodent parasite genomes missing orthologs for more than 730 *P. falciparum* genes (147, 148). In addition, there are differences in the both the liver and blood stages of infection. While the mouse parasites, *P. berghei* and *P. yoelii*, emerge from the liver after 2–3 days of infection, the human parasites, *P. falciparum* and *P. vivax*, require 7–10 days of pre-erythrocytic stage development (149). The formation of dormant pre-erythrocytic stages in *P. vivax* infections, which is a hallmark of *P. vivax* infections (150), is also not present in *P. berghei* and *P. yoelii* infections in mice, although liver forms of *P. yoelii* have been observed in the liver of their natural host, *Thamnomys gazellae*, at least 8 months post-sporozoite infection (151). Lastly, most murine studies are performed with genetically homogenous inbred mice with a limited MHC gene repertoire, which do not mimic the large genetic diversity of the human population. Laboratory mice are maintained in clean specific-pathogen—free facilities, hence the absence of the effect of environment (e.g., microbiome) on the mouse immune system may bias infection and vaccine studies (152). Taken together, while it is a powerful experiment tool, the traditional mouse model is not an ideal model, especially for studying pre-erythrocytic malaria vaccines.

### Non-human Primates

Due to the limitations of the traditional mouse models, there have been substantial efforts to develop alternative animal models that are able to generate adequate parallels in an *in vivo* approach of the human immune system. Historically, non-human primates (NHP) have been used as the alternative model. Compared to the mouse, NHP share a lot more similarities with the human. Simian *Plasmodium* species can infect various NHP species. In particular, human parasites can be adapted to NHP and some NHP can even support direct infection with *P. falciparum* and *P. vivax* (153). The *Aotus* monkeys have served as a valuable model. They can be infected by *P. falciparum* and *P. vivax* (154, 155). The NHP models are particularly important for assessments of *P. vivax* pre-erythrocytic stage vaccines (156), due to the formation of dormant pre-erythrocytic stages in *P. vivax* infections, which cannot be recapitulated in the mouse models. Simian malaria parasites, such as *P knowlesi* or *P. cynomolgi* in macaques, have also been used for immune and vaccine studies (157, 158). Vaccination with live sporozoites under chloroquine prophylaxis has shown promising efficacy data in Toque monkeys immunized with *P. cynomolgi* (159). However, the lack of availability, the high costs to maintain a colony and the restriction of utilization due to ethical issues limit the use of NHP, especially where large numbers are required.

### Humanized Mouse

In the more recent years, the development of a humanized mouse as an animal model (160, 161) has greatly facilitated the study of human malaria research. These models mainly arise from the xenotransplantation of human hepatopoietic cells and/or tissues, allowing the long-term establishment of components of human immunity in permissive immunodeficient mice.

Using a human liver chimeric SCID/Alb-uPA mouse, studies on the pre-erythrocytic stages can be performed (162, 163). While it has been shown to be a viable model to study *P. falciparum* pre-erythrocytic stage development, the study of human malaria in this model is limited to the pre-erythrocytic stage as *P. falciparum* cannot transit from the pre-erythrocytic stages to the blood stages in this model (164). Other drawbacks include infertility of the mice due to the SCID/Alb-uPA immunodeficient background (165), and hepatotoxicity and high neonatal mortality due to the uPA transgene expression (166). These have made the generation of large number of the mice extremely costly and difficult.

Due to these drawbacks, an alternative, a human liver chimeric FAH^−/−^Rag2^−/−^IL2Rγ^null^ (FRG) mouse, has been developed. These mice can be bred relatively easily and do not suffer from hepatotoxicity. In addition, this model has been shown to support robust pre-erythrocytic stage infection and development (149). When human RBCs were transplanted into these mice, the new model supported the transition from a pre-erythrocytic infection to a blood stage infection (167). The NOD mice deficient for the IL2Rγ gene and transgenic for the thymidine kinase gene (TK-NOG) is another model that has also been developed. These mice do not suffer from liver failure. A transient injection of the drug gancyclovir induces a controlled ablation of the mouse hepatocytes. Treated mice are easily repopulated with human hepatocytes (168). These mice can also be doubly engrafted with human red blood cells and this allows the full development of *P. falciparum* in the liver and the transition to the blood stage. Interestingly, this model also supported the liver stage development of another human parasite, *P. ovale* (169).These findings are extremely encouraging as this raises the possibility of using these models to study a liver stage infection and also a combined liver and blood stage infection. While the use of the humanized mice offers many new possibilities to study human malaria biology in a non-human model *in vivo*, it is worth noting that these mice are immuno-compromised, which makes them unsuitable for vaccine immunogenicity and efficacy studies. Nonetheless, they have shown to be useful in passive transfer experiments to test antibody efficacy (170–172).

Studies on the development of humanized mice with a fully reconstituted immune system are underway (173). In fact, humanized mice that possess the human immune system (HIS) have been established for malaria research, using recombinant adeno-associated virus (AAV)-based gene transfer technologies (174). With functional human CD4 T cells and B cells (HIS-CD4/B mice), these HIS mice were able to produce a significant level of human IgG against *P. falciparum* CSP upon immunization (175). The HIS-CD4/B mice were also protected against infection from an *in vivo* challenge with transgenic *P. berghei* sporozoites expressing the PfCSP protein following immunization. While these models are essential pre-clinical models to understand immune responses against human malaria, it is worth noting that these HIS mice still retain mouse myeloid compartments that are likely to influence antigen presentation and immune cell residency, and *in vivo* vaccine efficacy can only be examined using transgenic rodent malaria parasites expressing selected *P. falciparum* proteins. New iterations of humanized mice that possess the humanization of the liver, bone marrow, lymphoid compartments, and human erythrocytes would be the ideal mouse model and would greatly help to understand human malaria parasite infection and immunology. It would be an essential tool in providing a more accurate initial assessment of the safety profile and vaccine efficacy of malaria vaccine candidates before moving onto human studies.

### Human Volunteers

The most relevant model is the human host itself. The establishment of the CHMI model has greatly helped malaria research. The CHMI model involves exposing healthy human volunteers to the parasite *via* infective mosquito bites, monitoring the volunteers closely for signs and symptoms of malaria infection, and treating the volunteers with drug upon detection of fever and/or detection of parasites (45, 176).

The CHMI model uses the most relevant host-parasite pair. While CHMI has been performed *via* other routes such as intravenously and intramuscularly, it is more commonly done *via* infective mosquito bites. The use of infective mosquito bites in the model mimics the natural route of infection, offering advantages in the prediction of the potential efficacy of vaccine candidate against natural infections. However, it also has its limitations. The CHMI model is often performed with one parasite strain, while there are many antigenically diverse heterologous parasites in the field. Infection in the CHMI model is controlled and the parasite load is administered at one single time, whereas high parasite load at one single time is uncommon in natural field setting. Despite the limitations, CHMI studies with no observed efficacy could halt the pursuit of large and expensive clinical trials in malaria-endemic areas in time. CHMI studies with partial efficacy could provide insights on how protective efficacy could be improved by alterations in vaccination regimes such as the number of doses and number of immunizations. In particular, through a series of CHMI studies, the company Sanaria was able to optimize their PfSPZ vaccine, which is composed of radiation-attenuated, aseptic, purified, cryopreserved *P. falciparum* sporozoites, to induce sterile protection against homologous challenge for at least 59 weeks (24) and heterologous challenge for at least 33 weeks (51) in malaria-naïve individuals. The vaccination also prevented naturally transmitted heterogeneous *P. falciparum* in malaria-endemic adults in Mali for at least 24 weeks (vaccine efficacy of 29%) (14). Further CHMI studies to optimize dosage and vaccination regimes could improve the vaccine efficacy.

### Immunity and Correlates of Protection Against the Pre-erythrocytic Stage

The malaria parasite has a complex life cycle. Depending on the stage of development in its mammalian host, the parasite can be extracellular or intracellular. They can also infect different cell types. Hence, various innate and adaptive immune mechanisms are needed for parasite control and elimination. In order to develop an efficacious pre-erythrocytic stage vaccine, it is important to know the protective immune mechanisms to induce (Table 1).

**Table 1:** Immunity against pre-erythrocytic stage parasites.

**Immune response**	**Mode of action**	**References**
Innate type I interferon response	*Plasmodium* RNA as a pathogen-associated molecular pattern (PAMP) to activate a type I IFN response, which in turn reduce the liver parasite load	(177–179)
Cytokines	IFNγ induces the inducible nitric oxide enzyme to produce nitric oxide to kill the sporozoites	(180–182)
	TNFα increases the capacity of monocytes and macrophages to phagocytose parasite to limit parasite infection	(183, 184)
NK cells	Inhibit liver stage development to limit liver parasite load CD4 T cell priming	(185–187)
γδ T cells	IFN-γ production Inhibit intrahepatic parasitic development Prime CD4 and CD8 T cell responses	(188–194)
CD8α dendritic cells	CD8 T cell priming	(195–197)
CD8 T cells	Lysis of infected hepatocytes by perforin and granzymes	(184, 198–200)
	Indirectly through the action of pro-inflammatory cytokines such as IFN-γ to mediate anti-parasite effects	(180–182, 201–204)
CD4 T cells	B cell development to produce antibodies against the liver stage	(198)
	Survival of protective effector and memory CD8 T cells	(205, 206)
	Can be induced to expresses CD107a, a marker for cytotoxic degranulation, to mediate protection	(48)
Antibodies	Inhibit sporozoite motility in the liver Mediate cytotoxicity against sporozoites in the host skin	(207, 208)
	Opsonize sporozoites for the subsequent sporozoite phagocytosis by monocytes or macrophages	(208, 209)
	Inhibit sporozoite invasion into hepatocytes	(210)
	Inhibit sporozoite development inside the hepatocytes	(210)
	Bind to parasite neo-antigens such as heat shock protein expressed at the surface of infected hepatocytes to induce liver parasite killing through an antibody-dependent cell-mediated mechanism	(211)

In addition, through better understanding of the mechanisms involved in the protection, we could potentially identify correlates of protection. The identification of correlates of protection is particularly important to the vaccine development as it helps to assess vaccine efficacy and design better immunogens. Through various animal models, we are beginning to tease out the potential correlates of protective immunity.

### Innate Immunity

Upon infection, the innate immunity is triggered by the malaria parasites. Immune responses initiated by the innate immune system in response to parasites play key roles in protective immunity development. Early pro-inflammatory responses regulate anti-parasitic Th1 development and promote effector cell function for efficiently clearing infections. The use of a proper adjuvant is necessary to trigger the adequate innate pathway.

#### Cytokines

Cytokines play an important role in the protection against malaria. Upon infection, the RNA of the parasites is recognized by the cytosolic pathogen-recognition receptors of mouse hepatocytes. As a result, type I interferon pathway is induced, which can inhibit late stage parasites. Type I interferon leads to the recruitment of leukocytes that inhibit late liver forms through IFNγ (179, 212). IFNγ can inhibit the development of *P. yoelii* and *P. berghei in vitro* and *in vivo* in mice (177, 178), *P. falciparum* in human hepatocytes *in vitro* (213) and *P. vivax* infected chimpanzees *in vivo* (214). The effect of IFNγ is through the induction of the inducible nitric oxide synthase enzyme in hepatocyte which generates high of toxic nitric oxide (180–182).

In addition to IFNγ, IL6, and TNFα have also been implicated in protection. TNFα is able to inhibit parasite liver stage indirectly through induction of yet-to-be-identified mediators secreted by hepatocytes (215) or through IL-6 on non-parenchymal cells (216). IL-6 inhibits liver stage development through the induction of iNOS (217, 218).

#### NK and NKT Cells

NK and NKT cells are abundant in the liver, and interact with the parasites to initiate liver-stage cell-mediated immunity. Following the activation of the type I interferon pathway, the hepatocytes produce chemokines to recruit macrophages, neutrophils, lymphocytes, NK and NKT cells to the site of infection in mice (179, 219). This eventually leads to the killing of late liver stage parasites by NKT cells. NK cells have also been shown to inhibit the development of the liver stages in the hepatocytes, limiting the infection and/or reinfection in mice (185). NK cells also play an important role in CD4 T cell priming during murine malaria infections (186, 187), bridging between the innate and adaptive immunity.

#### γδ T Cells

Similar to NK T cells, γδ T cells are innate-like T cells that have been postulated to bridge the gap between innate and adaptive immunity (220). Early production of IFNγ by γδ T cells was detected following *in vitro* exposure of *P. falciparum*-infected RBCs to PBMCs from malaria-naïve donors (188). In mice, γδ T cells induced by whole sporozoites vaccination can inhibit intrahepatic parasitic development (190). γδ T cells are also important for the induction of early immunity against malaria (191). γδ T cell-deficient mice immunized with irradiated sporozoites were more susceptible to liver stage infection 42 h following a sporozoite challenge (191). γδ T cells can also directly prime CD4 and CD8 T cell responses *in vitro* (192, 193).

Sterile protection in mice following vaccination with irradiated sporozoites requires γδ T cells (194). Without γδ T cells, protective CD8 T cell responses were impaired (194). γδ T cells have been postulated to act either as effector cells that operate in the absence of αβ T cells, or as accessory cells for appropriate protective responses from other cells (194). In humans, γδ T cells have also been shown to recognize malaria antigens and proliferate, conferring immunity against clinical malaria in children from Uganda (221). In addition to influencing the protective CD8 T cell response, γδ T cells can also influence cytokine production. Higher frequencies and higher cytokine production by γδ T cells correlate with protection against subsequent infection in children living in endemic settings (189, 222). Recent studies on irradiated sporozoite vaccines have shown that γδ T cells expanded in a dose-dependent manner in immunized malaria-naïve subjects (6, 24), and were associated with protection (24). Hence, γδ T cells could be a potential correlate of protection, and further studies to better define a most appropriate outcome to represent a measurable positive correlation of γδ T cells with protection would be advantageous for vaccine development.

#### CD8α Dendritic Cells

Early adaptive immunity is triggered as early as a few hours post an infective mosquito bite, with T cell activation being observed in the skin draining lymph nodes in the murine model (196). After dermal inoculation, a fraction of sporozoites actively migrates to the draining lymph nodes (195). There is a direct uptake of the parasites by lymph-node resident CD8α dendritic cells followed by CD8 T cell-dendritic cell cluster formation in the draining lymph nodes (223). CD8α dendritic cells are also shown to be essential for the development of the protective immunity induced by intravenous injection of irradiated sporozoites since mice depleted of these subsets are not protected against a sporozoite challenge (224, 225). A subsequent study showed that splenic but not liver CD8α dendritic cells are the main cells involved in effector parasite specific-T cell priming (226). It was also shown recently that monocyte-derived CD11c cells infiltrated the liver after infection, acquired parasite-derived antigens and primed protective CD8 T cells (227). The role and functions of other dendritic subsets is controversial and remains to be determined (199, 228, 229).

### Adaptive Immunity

As with any vaccination, the focus has been to trigger the adaptive immunity to induce an efficacious and long-lasting immunity. Various arms of the adaptive immunity are required to act in concert to provide protection against malaria.

#### CD8 T Cells

CD8 T cells have been implicated as the principal effector cells, central to protection against malaria. The importance of CD8 T cells in protective immunity was first demonstrated in mice vaccinated with irradiated sporozoites (177). The sterile immunity induced by the vaccination was abolished when CD8 T cells were depleted (177, 178). CD8 T cells can kill the parasites in mice (200) either directly through lysis of infected hepatocytes by perforin and granzymes (184, 230) or indirectly through IFNγ-mediated protection (180–182, 201–204). It must be stressed that while leukocytes and, in particular, CD8 T cells can kill liver parasites by these mechanisms, they differ depending of the host/parasite combinations (231).

Given the central role of CD8 T cells in protection, it is one potential correlate of protective immunity. In humans, CD8 T cells have been shown to be associated with protection from severe malaria (232), and a few of the identified CD8 T cell responses are directed against pre-erythrocytic stage antigens such as LSA1 and CSP (233, 234). However, vaccination studies, where human volunteers were immunized with irradiated sporozoites, showed that, while CD8 T cell response were also detected against various pre-erythrocytic stage antigens, the responses were not found to be associated with protection (235). More recently, a human trial where human volunteers were immunized with irradiated sporozoites showed seemingly contradicting data, where sterile protection correlated with the numbers of IFNγ-producing CD8 T cells in isolated PBMCs (6). This is also evident in animal studies on irradiated sporozoite vaccines, where high frequency of parasite-specific CD8 T cells was observed in the liver of non-human primates and mice, and was associated with protection in mice (236). The differences are likely due to the differences in the vaccination regimes and the methods to detect/quantify T cells. Indeed, in humans, T cell activity is measured in peripheral blood, whereas, in mice, T cell activity is usually measured in spleen or liver.

##### Central memory T cells

Memory T cells provide long-term protection. Upon re-infection, these cells rapidly gain effector functions including cytokine production and lytic activity. There are three subsets of memory T cells: (1) central memory T cells, which predominantly reside in lymphoid tissues, (2) effector memory T cells, which reside in the spleen and peripheral tissues, and (3) tissue-resident memory T cells, which reside in the tissues and do not recirculate. The role of central memory T cells in protection against malaria is limited. While central memory T cells can produce IFN-γ after *in vitro* stimulation (201), their presence has not been associated with protection. Despite having a large proportion of central memory T cell, mice that were vaccinated with modified vaccinia ANKARA expressing the multiple epitope string and thrombospondin-related adhesion protein (ME-TRAP) were not protected from malaria challenge (237).

##### CD8 effector memory T cells

In contrast to the central memory T cells, the presence of effector memory T cells has been associated with sterile protection in the murine model, although large numbers are required for protection against malaria (238). Long-term sterile protection was only observed in mice when the parasite-specific CD8 T cells made up >1% of the total peripheral blood CD8 T cell population (238). Degree of protection in mice correlated with the frequencies of CD8 effector memory T cells present in liver, and failure to achieve the protective threshold frequency of these cells might make the host susceptible to infection (203).

##### CD8 tissue-resident memory T cells

More recently, a new subset of memory T cells, with a distinct gene expression profile, has been characterized (202). The liver tissue-resident memory T cells develop naturally during the course of an immune response following TCR stimulation, with rapidly expanding population due to the liver infection or inflammation in mice (239). These cells are found to be patrolling within the liver sinusoids, a process dependent on LFA-1–ICAM-1 interactions (240). Tissue-resident memory T cells were essential for sterile protection against sporozoite infection in mice following immunization with irradiated sporozoites (241).

#### CD4 T Cells

In contrast to CD8 T cells, the role of CD4 T cells in protection against malaria is not well understood. Despite this, it is clear that the development and maturation of an effective CD8T cell response is dependent on CD4 T cells help. CD4 T cells are activated to amplify the anti-pathogen response by driving B cell germinal responses and supporting CD8 T cell activation. Mouse hepatocytes express MHC Class I and Class II molecules that can be loaded with parasite antigen-derived epitopes following the TAP or the endosomal pathways (242–245). CD4 T cells are required to prime effective immunity. CD4 T cells recognizing CSP have been shown to protect against a *P. yoelii* sporozoite challenge in mice (243, 246). Pre-immunization but not pre-challenge depletion of CD4 T cells also resulted in a loss of protection in mice immunized with sporozoites, suggesting that CD4 T cells might provide signals for efficient maturation of effector CD8 T cells (247). In mice, CD4 T cells were essential to ensure survival of protective effector and memory CD8 T cell induced by irradiated sporozoites (205, 206). In humans, many studies have described a CSP-specific CD4 T cell response that is associated with protection against natural infection and disease (248), and is able to inhibit pre-erythrocytic stage development (249). CD4 T cells have also been shown to correlate with sterile protection in humans following immunization with live sporozoites under chloroquine prophylaxis. CSP-specific CD4 T cells were induced to express CD107a, a marker for cytotoxic degranulation, after immunizations with live sporozoites under chloroquine prophylaxis in humans, and these cytotoxic markers has been shown to be associated with sterile protection against the pre-erythrocytic stages (48). In addition to being crucial for B cell development to produce antibodies, CD4 T cells are also important for CD8 T cell responses.

While it is clear that CD4 T cells are involved in protective immunity against malaria, its use as a potential correlate of protection needs further validation. Together with CD8 T cells, the definitive role of CD4 T cells in protection requires more unraveling and the information will be critical to the development of a validated T cell-based correlate of protection for vaccine efficacy assessment.

#### Antibodies

Lastly, in addition to inducing an effective CD8 T cell response, the development of many malaria candidate vaccines also aims at being able to induce an effective antibody response. Antibodies are often the first host immune response being studied. Antibodies against the pre-erythrocytic stage can mediate protection by limiting pre-erythrocytic stage infection and development. More specifically, the antibodies do so by (1) inhibiting sporozoite motility in the dermis and liver (207), (2) mediating cytotoxicity against sporozoites in the host skin (208, 250), (3) opsonizing the sporozoites and subsequently facilitating sporozoite phagocytosis by monocytes or macrophages in the spleen or the liver (208, 209), (4) inhibiting sporozoite invasion into hepatocytes (210), (5) inhibiting sporozoite development inside the hepatocytes (210), and (6) binding to parasite neo-antigens such as heat shock protein expressed at the surface of infected hepatocytes and eventually inducing liver parasite killing through an antibody-dependent cell-mediated mechanism that is likely to involve Kupffer cells or NK cells (211).

Antibodies are potential correlates of protection. Passive transfer of RTS,S-induced human anti-CSP monoclonal antibodies into humanized mice at concentrations within the range observed in human, protected the mice against *P. falciparum* challenge (170). Immunization with genetically-attenuated sporozoites that arrest late in the liver stage development elicited protection against both a sporozoite challenge and a direct blood stage challenge by inducing the production of stage-transcending protective antibodies in mice (251). Sporozoite-specific antibodies induced by vaccination with irradiated sporozoites (252) or genetically-attenuated sporozoites (253) have also been shown to inhibit sporozoite invasion into human hepatocytes *in vitro* and correlate with protection in human individuals (254–256). However, a recent human study examining protection following vaccination with irradiated sporozoites in malaria-naïve individuals has found no significant correlation of antibody response with protection (46). In addition, there is no distinct antibody profile that allows differentiation of protected individuals from the susceptible individuals (257) following RTS,S vaccination. There is increasing awareness that, in addition of high level of antibodies, the quality of the antibodies is also important. *In vitro* assays to examine the functionality of the induced antibodies following vaccination of pre-erythrocytic stage vaccine have been developed. These assays include gliding motility assays (258), sporozoite traversal and invasion inhibition assays (6, 253, 259), and pre-erythrocytic stage development inhibition assays (136, 260). Recently, human monoclonal antibodies have been derived from volunteers immunized with irradiated sporozoites. These antibodies recognized an important epitope at the junction of the N terminal part and the repeat regions of the CSP. This can lead to the design of better CSP-based vaccines (146, 261). Further studies to draw parallels between the readouts of these assays and the protection in the field are necessary to develop a validated antibody-based correlate of protection for vaccine efficacy assessment.

## Concluding Remarks

In contrast to the limited efficacy of RTS,S and other subunit vaccines, vaccination with sporozoites has had more success. In addition to promising efficacy data, a series of recent clinical trials on sporozoite-based vaccines has demonstrated formidable advances in overcoming issues in vaccine manufacturing and delivery (6, 14, 23, 46). Furthermore, there are now data showing that sporozoite vaccines are safe and tolerated in malaria-endemic areas (14, 23). While the vaccine efficacy is markedly reduced against heterologous CHMI (as compared with homologous CHMI), it is encouraging that it offers some protection against heterologous CHMI (25). Further studies to optimize the immunization regimen could potentially improve the vaccine efficacy. Here, we reviewed the various types of vaccination strategies with sporozoites and the different animal models being used for the vaccination studies. We also discussed the mechanisms of protection against the pre-erythrocytic parasites. While the mechanisms of protection are slowly being unraveled, the establishment of validated correlates of protection for assessment of vaccine efficacy has proved to be challenging. Half of the world population is at risk of a malaria infection. The target population is highly diverse, with individuals from different age groups (infants, adults and elderly), different exposed status (endemic and non-endemic), and different immunological background (immunocompromised and pregnant). The presence of co-infections in some populations in malaria-endemic regions adds further complexity. In addition, the complexity of the parasite and the diversity of its genome also makes it difficult to definitively establish correlates of protection. Depending on which part of the parasite life cycle the malaria vaccine candidates target, different forms of immunity are induced. As it is still unclear if the ultimate goal of a malaria vaccine should be to protect against infection or simply to protect against disease, different clinical endpoints have been used to measure vaccine efficacy. Vaccine-induced immune responses that correlate with protection against one endpoint may not necessarily correlate with protection against a different endpoint. Hence, until date, there is no validated correlate of protection. A concerted effort to develop/refine relevant animal models, investigate the definitive mechanisms of protection and identify validated correlates of protection would greatly help to inform critical decisions in human vaccine clinical trial, which will accelerate future progress in the development of an efficacious malaria vaccine.

## Author Contributions

All authors listed have made a substantial, direct and intellectual contribution to the work, and approved it for publication.

### Conflict of Interest Statement

The authors declare that the research was conducted in the absence of any commercial or financial relationships that could be construed as a potential conflict of interest.
